# Factors related to infertility in Brazil and their relationship with success rates after assisted reproduction treatment: an integrative review

**DOI:** 10.5935/1518-0557.20200051

**Published:** 2021

**Authors:** Karina da Silva Maranhão, Maria Eduarda Gomes de Sena Melo Mariz, Esther Alice Dantas de Araújo, Gabriel Ribeiro de Souza, Karinna Veríssimo Meira Taveira, Danielle Barbosa Morais

**Affiliations:** 1 Department of Morphology, Federal University of Rio Grande do Norte, Natal, RN, Brazil

**Keywords:** male and female infertility, technologies of assisted reproduction, *in vitro* fertilization, intracytoplasmic sperm injection

## Abstract

This integrative review evaluated the most commonly diagnosed causes of infertility in men and women in Brazil, as well as the medically assisted reproduction technologies regularly employed in these cases. We searched in four electronic databases (PubMed, including Medline; Scopus; Web of Science and LILACS), and two grey literature (Google Scholar and OpenGrey), guided by the focused question: "What are the main factors responsible for male and female infertility in Brazil, and what are its relationships with success rates after assisted reproduction treatment?". We included interventional or observational studies, without limitation by language or year of publication. Our searches in the electronic indexers recovered 1,119 articles, and after analyzing the inclusion and exclusion criteria, 27 articles composed the body of analysis for this review. We grouped the studies into four themes: factors responsible for male and female infertility, assisted reproductive technologies (ART) used in the infertility treatment, assisted reproduction procedures, and clinical predictors of success rates in ART. Despite the scarcity of studies analyzing the association between infertility and assisted reproductive technologies in Brazil, it was possible to infer that the most prevalent infertility cause in women was endometriosis, while in men it was azoospermia. The most widely assisted reproductive technology applied in the country is the intracytoplasmic injection of spermatozoa (ICSI), ensuring better success rates in the treatment of infertility for men and women.

## INTRODUCTION

The World Health Organization (WHO) defines infertility as the inability of a couple to establish a clinical pregnancy after 12 months of regular unprotected sexual intercourse. Although commonly perceived as a female disorder, male factors are equally prevalent when considering the causes of infertility. Thus, it is estimated that female factors account for 35 to 40% of the causes of infertility, male factors for 20 to 40% and factors in which both have dysfunctions represent 20 to 30%, as the other idiopathic causes, causing sterility without apparent cause (Nardelli *et al*., 2014; WHO, 2019; Zeqiraj *et al*., 2018).

Considering the treatment of infertile patients, assisted reproduction becomes an important alternative to try to make pregnancy possible to couples that have difficulty in conceiving naturally. Fertilization rates in Brazil show that the country's assisted reproduction services are effective, reaching international standards. Data from the 12^th^ report of the National System of Embryo Production (SisEmbrio) indicate that the average success rate in fertilization is 76%, which is within the quality standards suggested in the international literature, ranging from 65% to 75% (SART, 2019; SisEmbrio, 2019). 

Although intracytoplasmic sperm injection is one of the most widely used treatments, assisted reproductive techniques also include conventional *in vitro* fertilization, artificial insemination and methods for monitoring reproductive cycles; and the method or technique to be used is chosen according to the patient's clinical condition. However, little is known, about the most prevalent causes of infertility in Brazil and which treatment is the most appropriate for each case. Considering the importance of this knowledge to support decision making during the planning of an assisted reproduction treatment, the objective of this integrative review was to answer the focused question: "What are the main factors responsible for male and female infertility in Brazil, and its relationship with success rates after assisted reproduction treatment?"

## MATERIAL AND METHODS

This integrative review covered the following stages: establishing the hypothesis and objectives of the integrative review; establishing the criteria for paper inclusion and exclusion (sample selection); defining the information to extract from the selected papers; evaluation of the studies included; interpretation of the results and synthesis of the studies. We used the PICO strategy to formulate the question addressed in this review, which is an acronym for Patient (or Population), Intervention, Comparison and Outcomes (Higgins *et al*., 2013).

This strategy allowed the identification of keywords, which helped locate relevant primary studies in the databases. Thus, the question that outlined the study was "What are the main factors responsible for male and female infertility in Brazil, and what is its relationship with the success rates after assisted reproduction treatment?" So, based on this question, the first element of the strategy (P) consists on patients with infertility; the second (I) refers to the assisted reproduction treatments; the third element (C), refers to the comparison of the interventions applied to the patients, and the fourth element (O) deals with the technique's effectiveness.

### Inclusion criteria

The inclusion criteria for the paper selection were: primary studies, conducted in Brazil; that cited infertility; which assisted reproduction treatment was employed; and the treatment outcome (whether there was pregnancy or not). There was no restriction regarding the time of publication or language.

### Exclusion criteria

We excluded papers based on the following criteria: (1) studies with animals; (2) treatments conducted in other countries; (3) studies that did not mention the infertility condition; (4) studies that did not mention the assisted reproductive technique employed; (5) studies that did not present the outcome of the treatment; (6) literature reviews, summaries, books, chapters of books, letters, opinion article, technical papers and guidelines.

### Information sources

We conducted a computerized bibliographic search in four databases: PubMed (including Medline), Scopus, Web of Science and LILACS (Latin American and Caribbean Literature in Health Sciences), and two grey literature: Google Scholar and OpenGrey, for any references that might could have been missed. Additional information on search strategies is provided in Appendix 1. All searches were conducted on April 14, 2019.

### Search

We combined the main descriptors related to the subjects we investigated, listed below, using the Boolean operators "AND" and "OR": "Assisted Reproductive Technology" OR "Assisted Reproductive Technologies" OR "Assisted Reproductive Technique" OR "Assisted Reproductive Techniques" OR "Assisted Reproductive Technic" OR "Assisted Reproductive Technics" AND "Brazil" (more details in Appendix 1).

We chose these keywords because they are associated with the object of study, and they belong to the catalog of PubMed descriptors (MESH Terms). We used the same keywords in LILACS, adding in this case their translations into Portuguese and Spanish, according to the Health Sciences Descriptors (DeCS).

We checked the references we obtained through the electronic search, and we removed the duplicates using the EndNote Web software (Thompson Reuters).

### Study selection and data collection process

In the first phase, four independent reviewers selected the studies (K.S.M., M.A.G.S.M., E.A.D.A. and G.R.S.), they evaluated the titles and summaries of the articles we identified by the search strategy. At that stage, we sorted the articles according to the eligibility criteria established. We retained for further analysis of the full text those articles that generated questions and were potentially eligible. In the second phase, we fully read the studies selected by the four reviewers, which made it possible to exclude other articles because they did not meet the revision proposal. In the third phase, we extracted the main information of the articles and synthesized in summary tables, so that they could guide the critical analyses of the studies selected. We resolved the disagreements by discussion, consultation and guidance of a fifth reviewer (D.B.M.).

### Data items

The data we collected included the year of publication, place of study/treatment, type of study (observational or interventional), infertility condition of the patient (men and women), assisted reproductive techniques employed in each case and the outcome of the treatment (if there was implantation, gestation and live births).

### Data synthesis

We calculated the prevalence of each infertility condition dividing the number of times in which each condition was cited in the articles by the total number of conditions presented in this review, multiplying the result by 100.

## RESULTS

### Result selection

**Figure 1:**
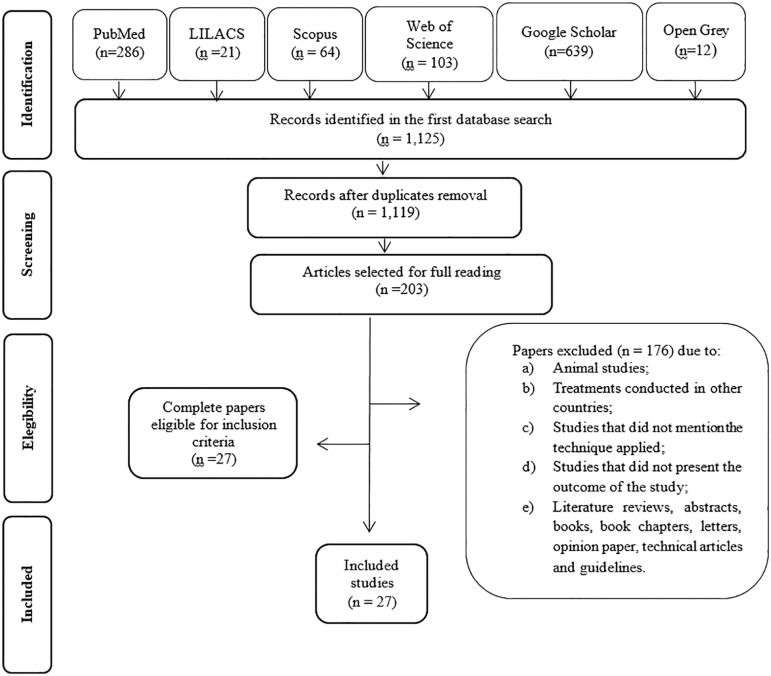
Flow diagram of literature search and selection criteria

Figure 1 depicts the bibliographic search process and the review selection criteria. The searches in the electronic databases and in the grey literature recovered 1,125 papers. After removing the duplicates, we evaluated 1,119 papers, and we excluded 916, resulting in 203 articles for full text reading. In this second step, we excluded 168 papers, because they did not meet the eligibility criteria. Thus, we included 27 remaining studies in the qualitative and quantitative synthesis.

### Characteristics of the studies

Table 1 shows a summary of the characteristics of the studies included in this review. Through the categorized analysis of the 27 articles that composed the final sample, we found that all were published between 2003 and 2019. Of these, 4 were cross-sectional studies (Pasqualotto *et al*., 2003; Esteves & Glina, 2005; Cota *et al*., 2012; Souza *et al*., 2017), 12 were cohort studies (Pinheiro *et al*., 2003; Borges *et al*., 2003; Glina *et al*., 2005; Pasqualotto *et al*., 2005; Romão *et al*., 2010; Semião-Francisco *et al*., 2010; Cota *et al*., 2012; Borges *et al*., 2013; Coelho Neto *et al*., 2015; Maia-Filho *et al*., 2015; Scheffer *et al*., 2017; Bercaire *et al*., 2018); 6 were case-control studies (Setti *et al*., 2011; Pasqualotto *et al*., 2012; Cavagna *et al*., 2012; Picinato *et al*., 2014; Donabela *et al*., 2015; Costa *et al*., 2016); 3 were case reports (Valle *et al*., 2012; Taitson *et al*., 2012; Borges *et al*., 2016) and 2 were randomized controlled trials (Geber & Sampaio, 2013; Nastri *et al*., 2013).

**Table 1:** Summary of descriptive characteristics and results of interest from the included studies (n=27)

*Author, year, place*	*Kind of study*	*Infertility condition presented by the patient*	*Assisted reproductive technique employed*	*Purpose of the study*	*Conclusion*
Bercaire *et al*., 2018, Brazil	Cohort	Advanced maternal age	IVF/ICSI	To evaluate a new protocol designed to improve POR through intra-ovarian androgenization, from the analysis of the number of recovered mature oocytes; fertilization, abortion and pregnancy rates.	The use of transdermal testosterone before the IVF cycle seems to increase the rates of recovered oocytes, pregnancy and births in patients with POR.
Borges *et al*., 2003, Brazil	Cohort	Obstructive azoospermia after vasectomy	ICSI	To evaluate the relationship between the period of post-vasectomy and the reproductive capacity post ICSI.	The interval between vasectomy and sperm recovery procedure has no effect on outcome until 14-year interval.
Borges *et al*., 2013, Brazil	Cohort	Leukocytospermia Tubal factor Idiopathic	IMSI	To compare IMSI results between cycles in which density gradient swim-up or centrifugation techniques were used for sperm preparation.	SUP and DGC techniques recovered improved sperm fractions and promoted similar IMSI results.
Borges *et al*., 2016, Brazil	Case-report	Oligozoospermia Astenozoospermia Teratozoospermia	ICSI	To compare ICSI results among groups with different mobile sperm count ranges, in couples with male infertility.	The total mobile sperm count is not a reliable predictor of ICSI result, because it does not consider morphology. Therefore, it was not able to predict ICSI laboratory or clinical results.
Cavagna *et al*., 2012, Brazil	Case-control	Leukocytospermia	IVF/IMSI	To analyze ICSI and IMSI results in couples in whom the male partner had leukocytospermia.	The results indicate that leukocytospermia may not have a negative effect on ICSI or IMSI cycle results.
Coelho Neto *et al*., 2015, Brazil	Cohort	Endometrioma Thin endometrium Poor ovarian response	IVF/ICSI	To analyze whether endometrial thickness and the presence of endometrioma are independent predictors of clinical pregnancy rate or simply associated with POR.	Both thin endometrium and the presence of endometrioma are associated with poor ovarian response, but are not important independent predictors of clinical pregnancy.
Coelho Neto *et al*., 2016, Brazil	Cohort	Endometriosis	IVF/ICSI	To evaluate whether women with endometriosis have different ovarian reserves and reproductive outcomes as compared to women without such diagnosis submitted to IVF and ICSI.	Women diagnosed with endometriosis are more likely to have deficient ovarian reserve; however, their chance of conceiving by IVF/ICSI is similar to that seen in patients without endometriosis and with a comparable ovarian reserve.
Costa *et al*., 2016, Brazil	Case-control	Idiopathic	IVF/ICSI	To investigate the genetic diversity of the HLA-G gene and its influence on infertility, and the result after assisted reproduction treatment in women with and without pregnancy success.	Some HLA-G alleles have been associated with reproductive failure.
Cota *et al*., 2012, Brazil	Cross-sectional	Endometriosis Ovary polycystic syndrome	IVF/ICSI	To investigate whether the prevalence of oocyte dysmorphism is influenced by the type of pituitary suppression used in ovarian stimulation.	In terms of oocyte morphology quality, there is no difference between the multiple dose protocol of the antagonist and the long-term agonist protocol.
Donabela *et al*., 2015, Brazil	Case-control	Endometriosis	ICSI	To compare the expression of SOD1, SOD2 and GPX4 in mature oocyte CCS from infertile women and investigate the interaction between the expression of these genes and clinical pregnancy.	Only infertile women with moderate/severe endometriosis had increased SOD1 expression incumuluscells, compared to women with minimal/mild endometriosis and controls, with positive interaction between increased expression and occurrence of clinical pregnancy.
Esteves & Glina, 2005, Brazil	Cross-sectional	Azoospermia	ICSI	To analyze if testicular histological patterns in a group of azoospermic men with varicocele are predictive of the result of treatment after correction of subinguinal microsurgical varicocele.	Microsurgical repair of varicocele in men with non-obstructive azoospermia may result in the appearance of spermatozoa in the ejaculate, when there is hypo spermatogenesis or cessation of maturation in the testicular histological diagnosis.
Geber & Sampaio, 2013, Brazil	Randomized controlled trial	Male factor	ICSI/IVF	To evaluate whether the prolonged action of GnRH in the luteal phase, in the assisted reproductive treatment cycles, affects the pregnancy rates according to the duration of its action in this phase.	Regardless the GnRH action duration in the luteal phase, there was no significant association with pregnancy rates.
Glina *et al*., 2005, Brazil	Cohort	Obstructive azoospermia	ICSI	To evaluate the rate of sperm recovery in each of the histopathological groups: hypospermatogenesis-hypo; maturation arrest on spermatogenesis-MA; and single Sertoli cell-SSO.	There were sperm cells in 33% of the procedures in patients with MA, 50% in patients with hypo and 40% of the procedures in patients with SSO.
Maia-Filho *et al*., 2015, Brazil	Cohort	Male factor Endometriosis Tubal factor Ovulatory factor Idiopathic	ICSI	To evaluate the expression of endometrial matrix metalloproteinases (MMPs) 2 and 9 and E-cadherin in the peri-implantation phase of infertile women undergoing IVF cycles.	MMP-2, MMP-9 and E-cadherin are expressed in the endometrium of infertile women during the receptive phase of the natural menstrual cycle. However, there is no correlation between the expression of these molecules and IVF clinical results.
Nastri *et al*., 2013, Brazil	Randomized controlled trial	Endometrial factor	ICSI	To investigate the effect of endometrial injury on reproductive outcomes and to evaluate the pain involved in the procedure for women undergoing assisted reproduction techniques.	The rates of implantation, birth and clinical pregnancy were higher in patients submitted to endometrial injury. However, there were reports of considerable pain during the procedure.
Pasqualotto *et al*., 2005, Brazil	Cohort	Azoospermia	ICSI	To evaluate the fertilization, pregnancy and miscarriage rates post- ICSI, with epididymal or testicular spermatozoa in different types of azoospermia.	Although there were no differences in the evaluated rates, fertilization and implantation, rates were higher in patients with congenital blockage of the seminal pathway. The rate of pregnancy was higher, and the rate of miscarriage was lower when epididymis sperm was used compared to testicular sperm.
Pasqualotto *et al*., 2003, Brazil	Cross-sectional	Obstructive azoospermia Vasectomy Bilateral absence congenital vas deferens	ICSI	To evaluate the effectiveness of repeated PESA procedures.	The procedures were effective in more than one-third of the repeated PESA attempts, resulting in the presence of mobile spermatozoa.
Pasqualotto *et al*., 2012, Brazil	Case-control	Varicocele	ICSI	To evaluate the effect of varicocelectomy on sperm quality and gestation rate after ICSI.	Although varicocelectomy should always be performed before assisted reproduction, this procedure does not increase pregnancy rates or decrease miscarriage rates after ICSI.
Picinato *et al*., 2014, Brasil	Case-control	Endometriosis Anovulation Tubal factor Idiopathic male factor	ICSI	To examine the effects of oocyte submission to PM before ICSI.	PM was associated with increased fertilization rates, and reduced cleavage rate and number of high-quality embryos.
Pinheiro *et al*., 2003, Brazil	Cohort	Male factor	ICSI	To determine implantation and pregnancy rates in patients submitted to ICSI and treated with beta2-adrenergic agonists, considering the uterine-relaxing action of these agents.	Pregnancy and implantation rates did not differ significantly between the groups. In addition, there were no significant differences in miscarriage rates.
Romão *et al*., 2010, Brazil	Cohort	Male factor Tubal factor Ovulatory factor Uterine factor	ICSI	To assess the impact of the average oocyte diameter on the occurrence of fertilization and embryonic quality in assisted reproduction cycles.	The diameter of mature oocytes does not appear to be related to fertilization or the quality of human embryos development on days 2 and 3 after ICSI.
Scheffer *et al*., 2017, MG/Brazil	Cohort	Advanced maternal age	ICSI	To evaluate embryonic quality associations and follicular reserve markers, such as age, FSH and AMH.	Age is predictive of the number of oocytes collected and of embryonic quality, factors that influences the outcome of assisted reproductive technologies.
Semião-Francisco *et al*., 2010, Brazil	Cohort	Azoospermia	ICSI	To compare ICSI results among patients with OA or NOA submitted to TESA and PESA procedures.	There were no statistically significant differences in clinical pregnancy or implantation rates among patients submitted to TESA and PESA.
Setti *et al*., 2011, Brazil	Case-control	Poor ovarian response	ICSI	To test the hypothesis that women of advanced age and with POR have an increase in embryo with chromosomal changes when compared to elderly women, who presented a normal ovarian response.	Women of advanced age and with POR to gonadotrophins are not at increased risk of producing aneuploid embryosin vitro.
Souza *et al*., 2017, Brazil	Cross-sectional	Poor ovarian response	IVF/ICSI	To investigate the impact of follicular washing on the number of oocytes recovered, oocyte maturity, rate of fertilization, embryonic development and pregnancy rate in POR women.	Follicular lavage may be an appropriate alternative to increasing the number of oocytes and pregnancy rates in patients with POR.
Taitson *et al*., 2012, Brazil	Case report	Obstructive azoospermia	ICSI	To evaluate the effectiveness of PESA in an azoospermic 81-year old patient.	The procedure was effective, with pregnancy and birth of two babies.
Valle *et al*., 2012, Brazil	Case report	Ovarian failure Idiopathic	ICSI	To demonstrate that human embryos can be successfully vitrified/heated twice in the cleavage phase.	Human embryos can be successfully vitrified/heated twice in the cleavage phase, being able to promote successful pregnancies and deliveries.

AMH: Anti-Mullerian Hormone; DGC: Density Gradient Centrifugation; FSH:Follicle-Stimulating Hormone; GnRH: Gonadotropin Releasing Hormone; HLA-G: G-Human Leukocyte Antigen; IVF: In Vitro Fertilization; ICSI: Intracytoplasmic Sperm Injection; IMSI: Intracytoplasmic Injection of Morphologically Selected Sperm; NOA: Non-obstructive azoospermia; OA: Obstructive azoospermia; PESA: Percutaneous Epididymal Sperm Aspiration; PM: Polarization Microscopy; POR: Poor Ovarian Response; SCO: Sertoli Cell-Only; SOD: Superoxide Dismutase; SUP: Swim-up; TESA: Testicular Sperm Aspiration.

**Figure 2:**
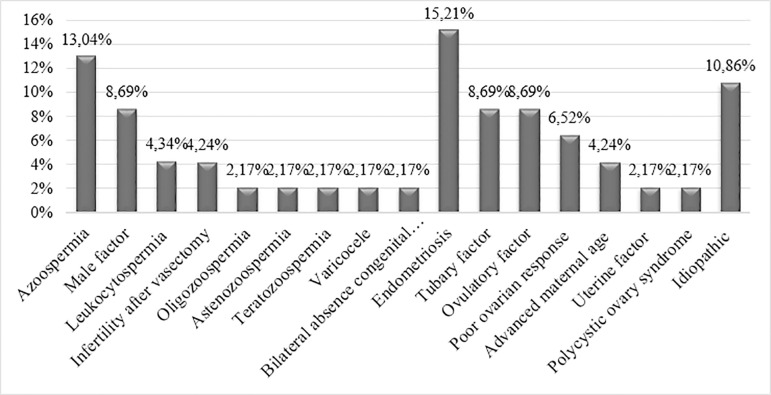
Causes of male and female infertility most frequently reported in this review

In 15.21% of the articles included in this review, endometriosis is cited as the main condition causing female infertility (Cota *et al*., 2012; Nastri *et al*., 2013; Maia-Filho *et al*., 2015; Picinato *et al*., 2014; Donabela *et al*., 2015; Coelho Neto *et al*., 2015; 2016); and in 13.04% azoospermia is the male infertility condition (Pasqualotto *et al*., 2003; Glina *et al*., 2005; Esteves & Glina, 2005; Pasqualotto *et al*., 2005; Semião-Francisco *et al*., 2010; Taitson *et al*., 2012). Figure 2 shows the other infertility conditions presented by the patients.

Regarding the assisted reproduction techniques, 7 studies (25.92%) mentioned that patients were submitted to intracytoplasmic sperm injection (ICSI) or conventional *in vitro* fertilization (IVF) (Cota *et al*., 2012; Geber & Sampaio, 2013; Coelho Neto *et al*., 2015; 2016; Costa *et al*., 2016; Souza *et al*., 2017; Bercaire *et al*., 2018); 18 studies (66.66%) cited ICSI exclusively (Pinheiro *et al*., 2003; Borges *et al*., 2003; Pasqualotto *et al*., 2003; Glina *et al*., 2005; Esteves & Glina, 2005; Pasqualotto *et al*., 2005; Romão *et al*., 2010; Semião-Francisco *et al*., 2010; Setti *et al*., 2011; Pasqualotto, *et al*., 2012; Valle *et al*., 2012; Taitson *et al*., 2012; Nastri *et al*., 2013; Picinato *et al*., 2014; Maia-Filho *et al*., 2015; Donabela *et al*., 2015; Borges *et al*., 2016; Scheffer *et al*., 2017); and 2 studies (7.40%) cited the Intracytoplasmic Injection of Morphologically Selected Sperm (IMSI) (Cavagna *et al*., 2012; Borges *et al*., 2013). IVF was used mainly in cases associated with female infertility, such as endometriosis, poor ovarian response, polycystic ovary syndrome and advanced age. ICSI cycles were associated with cases similar to IVF, and causes related to the reduction in sperm count and/or quality. IMSI predominated in leukocytospermia conditions.

## DISCUSSION

This integrative review investigated the main conditions of male and female infertility in Brazil, and their relationship with success rates after the treatment of assisted reproduction. By our knowledge, it is the first to address this theme, allowing the compilation about the relations between such conditions and the assisted reproduction treatments employed in the country.

### Main factors responsible for female and male infertility

The main factors associated with female infertility included endometriosis, tubal factor, polycystic ovary syndrome, endocrine/anovulatory and advanced age; while for males it included seminal alterations (azoospermia, oligozoospermia, asthenozoospermia and teratozoospermia), varicocele and infertility after vasectomy, and anatomical factors.

The most prevalent factor in women is endometriosis, which consists of having endometrial tissue outside the uterus, inducing a chronic inflammatory reaction and the formation of adhesions. In this pathology, the endometrial tissue is commonly lodged on the peritoneal surface, ovaries and rectovaginal septum. Endometriosis is increasing in women and has an important relationship with female infertility, since endometrial tissue even outside the uterus, continues to be stimulated monthly by the action of the menstrual cycle hormones. This leads to an inflammatory reaction, which consequently influences the hormonal regulation necessary for ovulation, as well as embryo implantation (Crosera *et al*., 2010; Nácul & Spritzer, 2010).

The peritoneal tube factor is also an important cause of female infertility. Pelvic inflammatory diseases, especially those caused by *Chlamydia trachomatis* and *Neisseria gonorrhoeae*, are undoubtedly among the most common causes of functional loss of uterine tubes, besides other causes such as endometriosis - which leads to tubal infertility (Fernandes *et al*., 2014).

Despite advances in assisted reproduction, poor ovarian response (POR) to gonadotrophin stimulation remains a problem in fertility treatment, especially in IVF, where a large number of oocytes are desirable. Many protocols and adjuvant therapies have already been proposed for the treatment of bad responders; among them ANDRO-IVF, a new protocol that promotes intraovarian androgenization. The idea that testosterone could be useful to improve poor ovarian response is based on studies which suggest that androgens play an important role in the early stages of follicular development. Besides that, the increase in intraovarian concentration of androgens appears to raise the expression of Follicle-Stimulating Hormone (FSH) receptors in granulosa cells, increasing ovary sensitivity to FSH. Transdermal testosterone before the IVF cycle seems to increase the rate of pregnancy and births by decreasing the required dose of gonadotropin, as well as the number of days of stimulation. Although these studies are still scarce, they have been promising, bringing new possibilities of adjuvant treatment to patients with low response to ovarian stimulation (Setti *et al*., 2011; Borges *et al*., 2013).

Polycystic ovary syndrome (POS) is the main gynecological endocrinopathy in women of reproductive age, and it is the most common cause of infertility due to anovulation. This endocrinopathy occurs in the ovaries from an imbalance in hormonal levels of androgens and insulin, causing the formation of ovarian cysts, which can interfere with the ovulation process. In this case, the patient does not ovulate properly and, therefore, may present long intervals between menstrual cycles, and may remain without menstruation for months, thus leading to infertility (Santana *et al*., 2008).

Advanced maternal age is also an important factor associated with female infertility, since natural fecundity and pregnancy rates decline with increasing age. This is due to significant reductions in the number and quality of the oocytes, since women are born with a limited pool of oocytes, which degenerate throughout their lives at each menstrual cycle. However, the main factor in the etiology of age-related female infertility is the decline in oocyte quality, since older women have higher rates of single chromatid abnormalities in oocytes, as well as chromosomal aneuploidies (Scheffer *et al*., 2017).

Azoospermia was the most prevalent factor in men, which consists of the absence of sperm in the semen. It is classified as obstructive or excretory azoospermia (OA), in cases where production is normal, but there is excretory pathway obstruction (epididymis, deferent or ejaculatory duct); vasectomy is the main example, and non-obstructive or secretory azoospermia (NOA), when there is testicular failure leading to the abolition of sperm production. In combination, there is azoospermia in approximately 10% of men with infertility (Harris & Sandlow, 2008; Vieira *et al*., 2009).

Seminal alterations, such as azoospermia (Pasqualotto *et al*., 2003; Glina *et al*., 2005; Esteves & Glina, 2005; Pasqualotto *et al*., 2005; Semião-Francisco *et al*., 2010; Taitson *et al*., 2012), oligozoospermia (Borges *et al*., 2016), asthenozoospermia (Borges *et al*., 2016) and teratozoospermia (Borges *et al*., 2016) were the most frequently cited infertility conditions in this review, and refer to abnormalities in sperm number, motility and morphology. Azoospermic men do not have sperm in their ejaculate, while in oligozoospermia there is a reduction in the amount of sperm present in the ejaculated fluid. In asthenozoospermia, there is a decreased spermatozoa motility, which can cause the inability or difficulty for sperm to fertilize the oocyte, while in teratozoospermia there is a large percentage of abnormally shaped sperm. There are morphology defects in different parts of the sperm: head, intermediate part and tail, which may render the natural fertilization, process impossible (Zegers-Hochschild *et al*., 2017).

Varicoceles are also an important cause of male infertility, being a condition that involves dilations and crooked veins of the pampiniform plexus, which drain blood from the testicles. Such alterations cause changes in temperature, oxygenation, nutrition and release of free radicals in testicular cells, which may compromise spermatogenesis and, consequently, cause male infertility (Cocuzza, 2011).

Another condition associated with male infertility is post-vasectomy infertility, a form of obstructive azoospermia, caused by the ligation of the deferent ducts in man. In these cases, men become infertile due to interruptions in sperm transit. However, given the widespread use of vasectomy as a method of contraception, there is a growing demand for vasovasostomy, which consists of surgical reversal, in which the two separate parts of the deferent duct are reconnected. ICSI combined with percutaneous epididymis sperm aspiration (PESA), or testicular sperm aspiration (TESA) is currently a widely used approach for fertility restoration in post-vasectomy cases, where anastomosis has failed. However, there is a relationship between the post-vasectomy period and the reproductive capacity of spermatozoa, that is, the shorter it is, the higher the chances of fertility return by ICSI. The rates of pregnancy and implants decrease significantly when sperm recovery is performed 14 years after surgery (Borges *et al*., 2003).

The articles included in this revision also mentioned infertility caused by anatomic factors, such as the congenital bilateral absence of the deferential duct, an anomaly responsible for approximately 6% of the cases of obstructive azoospermia, and for 1-2% of the cases of infertility in men (Bernardino *et al*., 2003).

### Assisted reproductive technologies most commonly used in infertility treatment

Five techniques stand out today in medically assisted reproduction: intrauterine insemination (IUI), IVF, ICSI and IMSI. IUI is a simple procedure of three steps: follow-up of natural follicular development, semen collection and processing and artificial insemination through a catheter. IVF, in turn, is based on the collection of gametes, which are placed in contact on the same culture plate, and the sperm will then fertilize the oocyte outside the female body. After embryo fertilization and cultivation in the laboratory, it is transferred to the uterus. ICSI is a variant of IVF, in which we select a spermatozoon and inserted it into the oocyte cytoplasm with the help of a micromanipulator, surpassing the radiate crown and the zona pellucida, thus increasing fertilization rates. Finally, IMSI or super ICSI, is a technique that carefully selects sperm based on morphology, using a microscope that enables a magnification higher than 6,000 times, while in ICSI this increase is of 400X, which, in the second case, does not enable the identification of some morphological abnormalities. However, IMSI is not used world widely anymore, because the technology offers no benefit over ICSI for live birth or miscarriage rates; although there is evidence that IMSI improves clinical pregnancy rates, this evidence is of very low quality (Santos, 2010; González-Ortega *et al*., 2010; Sermondade *et al*., 2011; Teixeira *et al*., 2020).

Among the AR technologies used in the country, ICSI is usually the main technique of choice, since it enables to solve the problems of infertility in most cases in which sperm quantity or motility is significantly reduced (Pasqualotto *et al*., 2003; Glina *et al*., 2005; Esteves & Glina, 2005; Pasqualotto *et al*., 2005; Semião-Francisco *et al*., 2010; Taitson *et al*., 2012). The greatest advantage compared to other techniques is that fertilization is less affected by concentration, motility and/or sperm morphology, and its disadvantage is the high cost of the procedure. Therefore, ICSI is today the most used technique in cases where there are serious changes in seminal parameters (Nagy *et al*., 1995; Santos, 2010).

Considering the success rates achieved after the treatments employed, IVF was more effective in cases of infertility caused by advanced or profound endometriosis; in women with absence of uterine tubes or tubal lesions that prevented natural fertilization or artificial insemination, and in cases of idiopathic infertility. On the other hand, ICSI was effective in cases similar to conventional IVF, and in cases of severe male infertility, obstructive or non-obstructive, leading to absence of sperm in the ejaculate; in cases of varicocele, and in cases using cryopreserved samples.

### AR treatment procedures and their relationship with success rates

In addition to these high complexity techniques, we employ several methods to obtain and process gametes, through complementary technologies and procedures that help increase gestational rates. We use sperm recovery methods, such as PESA and TESA, in conditions when the patient has azoospermia, either OA or NOA, with the goal of increasing fertilization rates in assisted reproduction treatments. These techniques are widely used in post-vasectomy infertility, when there is obstructive azoospermia, followed by ICSI, to achieve better success rates (Pasqualotto *et al*., 2003; Semião-Francisco *et al*., 2010; Taitson *et al*., 2012).

Sperm capacitation techniques, such as Swim-up (SUP) and density gradient centrifugation (DGC), which recovers fractions of high-quality sperm, are recommended to select spermatozoa with greater motility, by eliminating seminal plasma, which contains motility inhibitors, and immobile spermatozoa, along with immature cells and debris. Both sperm capacitating techniques are advantageous to be performed prior to assisted reproduction treatments, since the increase in spermatic motility favors penetration into the oocyte and, consequently, increases the fertilization rates (Borges *et al*., 2013).

On the other hand, follicular washing is a good alternative to enable a greater number of oocytes in patients with POR, since it improves the chances of overcoming the retention of oocytes in the follicle during direct aspiration or in the collection system, thus increasing the number of oocytes recovered (Souza *et al*., 2017).

Endometrial injury is an intentional damage to the endometrium by biopsy or curettage, which can induce decidualisation and increase the likelihood of implantation. This is because it induces a significant increase in the secretion of cytokines, interleukins, growth factors, macrophages and dendritic cells, which may be beneficial for the implantation of embryos and may lead to better synchronization between the endometrium and the embryo transfer, with a consequent increase in the rates of live births and clinical pregnancy. Overall, the results suggest a benefit from endometrial scratching. However, the studies have significant limitations; thus, the results may be biased. Therefore, it is not possible to say with any confidence whether endometrial injury can increase the probability of pregnancy. It is also important to consider potential adverse events of this procedure, including excessive pain and bleeding (Garris & Garris, 2003; Li & Hao, 2009; Lensen *et al*., 2016; Gnainsky *et al*., 2010; Nastri *et al*., 2012).

### Clinical predictors of success rates in assisted reproductive technologies

The search for predictors of success in highly complex assisted reproductive treatments, such as IVF and ICSI, is essential, since it can determine the efficiency of the techniques and, consequently, the rates of gestational success. Among the predictors of success in the high-complexity assisted reproduction treatments discussed in this review, those who best determined the effectiveness of the techniques, as well as the rates of gestational success were: age, embryo quality and number of oocytes recovered, followed by two promising predictors: the HLA-G allele diversity profile and the SOD1 gene expression.

Maternal age is an important clinical predictor of success rates, since it is correlated to the number of oocytes collected and embryo quality, factors that influence the outcomes of assisted reproduction treatments. As age progresses, natural fecundity and pregnancy rates decline, which occurs due to a significant reduction in the number and quality of oocytes. However, the main factor in the etiology of age-related female infertility is the decline in oocyte quality, since older women have higher rates of single chromatid abnormalities in oocytes, as well as chromosomal aneuploidies (Scheffer *et al*., 2017).

We analyzed embryonic quality from studying the morphology of embryos and the way they evolved, until the 5^th^ or 6^th^ day after IVF procedures. Some of the parameters we assessed were oocyte shape, cellular fragmentation degree, number of blastomeres and their morphology, the presence of vacuoles and the first polar body (1^st^ PB). Regarding quality, the following factors yield bad prognosis: embryos with irregular cleavages, anuclear fragments, irregular blastomeres, low number of blastomeres, and absence of 1^st^ PB, which in turn may indicate that the oocyte is still immature or that it has already become post-mature, both unfit for insemination. The indicators of good prognosis include the occurrence of cleavage at the right time (leading to a suitable number of cells for the day of development; for example, the presence of embryos with 4 cells on day 2 and with 8 cells on day 3); harmonic and balanced cell division (leading to blastomere symmetry); absence of embryonic fragmentation; vacuole-free cytoplasm and signs of compaction (Araújo *et al*., 2008).

However, even embryos classified as being of good quality will not necessarily result in a clinical pregnancy, since endometrial health will be the determining factor. This is because endometrial receptivity disorders represent a potential source of implantation failure, even with good quality embryos. Embryonic implantation is a complex process that requires a synchronization between a healthy embryo and a receptive endometrium. This integrative capacity depends on several endocrine, paracrine and autocrine factors, which are responsible for endometrial receptivity. This is the period during which the epithelium of the endometrium acquires function, even transient, for blastocyst welcoming and implantation. This period is of extreme importance for implantation, since if the embryo reaches the endometrium or is transferred during IVF in a period outside the implantation window (either before or after), implantation will not occur (Martins, 2007; Tan *et al*., 2018).

The number of oocytes recovered is also an important prognostic variable, since there is a correlation between this number and treatment success rates. In Brazil, the average number of oocytes produced per woman is about 9.3. However, higher chances of success are obtained from IVF cycles with 15 oocytes recovered, with success rates of 65% of live births in women aging 18-34 years; 50% in patients aging 35-37 years; 47% of live births for women with 38 or 39 years old; and 35% of live births for women aged 40 or over. However, even with the average number of oocytes recovered below the considered "ideal", the average fertilization in Brazil reaches 76% (Sunkara *et al*., 2011; SisEmbrio, 2019).

The human-G leukocyte antigen (HLA-G) encodes a protein class I (Ib), whose expression is intense in trophoblastic cells, and has therefore been recognized to confer immunological tolerance to the fetus. Some HLA-G alleles have been associated with increased or reduced expression of the levels of this protein, which have been associated with reproductive failure. However, it is still difficult to reach a consensus on the role of the different HLA-G alleles during pregnancy (Costa *et al*., 2016). However, it can be a promising clinical predictor, since identifying regulatory differences between alleles will not only improve our understanding of pregnancy biology, but it can also help unravel the immunogenic factors associated with infertility.

Although poorly explored, the gene expression evaluation in cumulus cells (CCS) of infertile women with endometriosis has been used to investigate the mechanisms involved in infertility associated with this disease. There is a greater expression of the SOD1 gene in infertile patients with moderate/severe endometriosis, when compared with healthy infertile patients or those with mild endometriosis. These results suggest an attempt to prevent oocyte oxidative damage triggered by the disease. Since only infertile women with moderate/severe endometriosis showed increased SOD1 expression in CCS compared to women with minimal/mild endometriosis and controls with positive interaction between increased expression and occurrence of clinical pregnancy, SOD1 may be a potential biomarker of clinical pregnancy, followed by ICSI (Donabela *et al*., 2015).

The birefringence analysis of the zona pellucida and visualization of the meiotic spindle under polarization microscopy (PM) are also clinical predictors, since they have correlation with implantation and pregnancy rates. These rates were significantly higher when transferring embryos derived from high refringence oocytes (HR), while miscarriage rates were higher when transferring embryos derived from low refringence oocytes (LR). Moreover, the fertilization rate was significantly lower when the meiotic spindle was not visible. However, a limitation of this technique is that while it may potentially increase these fertilization rates, this strategy may reduce the number of high-quality embryos, since the additional handling of the oocyte required to perform the PM is the main reason for this reduction (Picinato *et al*., 2014).

Despite the findings described here, this review had some methodological limitations, the main one being the scarcity of studies analyzing the association between infertility and assisted reproductive technologies in Brazil. Another possible limitation is that we did not include studies published on non-conventional and low-diffusion access bases, despite our effort using search strategies in a broad and careful way.

## CONCLUSION

The qualitative and quantitative analysis of the studies that associated infertility and assisted reproductive technologies in Brazil showed that the main factors of female infertility were endometriosis, tubal factor, polycystic ovary syndrome, endocrine/anovulatory and advanced age, while males had reductions in sperm count and/or quality, infertility after vasectomy, varicocele and anatomical factors.

Regarding post-treatment success rates, they were associated with the assisted reproduction technologies chosen for each infertility condition, as well as the use of procedures such as PESA, TESA, follicular lavage and endometrial injury, which may result in better gestational rates when employed during assisted reproduction cycles.

ICSI has been the predominant technique employed and has proven to be the most promising for the treatment of female and male infertility.

## Appendix 1

**Appendix 1:** Search keywords used in databases

*Database*	*Keywords*
Pubmed	"Assisted Reproductive Technology" AND "Brazil"; "Assisted Reproductive Technologies" AND "Brazil"; "Assisted Reproductive Technique" AND "Brazil"; "Assisted Reproductive Techniques" AND "Brazil"; "Assisted Reproductive Technic" AND "Brazil"; "Assisted Reproductive Technics" AND "Brazil".
Scopus	"Assisted Reproductive Technology" OR "Assisted Reproductive Technologies" AND "Brazil"; "Assisted Reproductive Technique" or "Assisted Reproductive Techniques" AND "Brazil" "Assisted Reproductive Technology" OR "Assisted Reproductive Technologies" AND "Brazil"; "Assisted Reproductive Technic" OR "Assisted Reproductive Technics" AND "Brazil".
Web of Science	"Assisted Reproductive Techniques" AND "Brazil".
LILACS	"Assisted Reproductive Technique" OR "Técnicas Reproductivas Asistidas" OR "Técnicas de Reprodução Assistida" AND "Brazil"; "Assisted Reproductive Techniques" OR "Técnicas Reproductivas Asistidas" OR "Técnicas de Reprodução Assistida" AND "Brazil"; "Assisted Reproductive Technic" OR "Técnicas Reproductivas Asistidas" OR "Técnicas de Reprodução Assistida" AND "Brazil"; "Assisted Reproductive Technics" OR "Técnicas Reproductivas Asistidas" OR "Técnicas de Reprodução Assistida" AND "Brazil"; "Assisted Reproductive Technology" OR "Técnicas Reproductivas Asistidas" OR "Técnicas de Reprodução Assistida" AND "Brazil"; "Assisted Reproductive Technologies" OR "Técnicas Reproductivas Asistidas" OR "Técnicas de Reprodução Assistida" AND "Brazil".
Google Scholar	"Assisted reproductive techniques" AND "Brazil".
Open Grey	"Assisted Reproductive Technique" OR "Assisted Reproductive Techniques" AND "Brazil"; "Assisted Reproductive Technic" OR "Assisted Reproductive Technics" AND "Brazil"; "Assisted Reproductive Technology" OR "Assisted Reproductive Technologies" AND "Brazil".
